# SASH1 is a prognostic indicator and potential therapeutic target in non-small cell lung cancer

**DOI:** 10.1038/s41598-020-75625-1

**Published:** 2020-10-29

**Authors:** Joshua T. Burgess, Emma Bolderson, Mark N. Adams, Pascal H. G. Duijf, Shu-Dong Zhang, Steven G. Gray, Gavin Wright, Derek J. Richard, Kenneth J. O’Byrne

**Affiliations:** 1 Cancer and Ageing Research Program, Centre for Genomics and Personalised Health at the Translational Research Institute (TRI), Queensland University of Technology, 37 Kent Street Woolloongabba, Brisbane, 4102 Australia; 2 Princess Alexandra Hospital, Ipswich Road, Woolloongabba, Brisbane, QLD 4102 Australia; 3 University of Queensland Diamantina Institute, The University of Queensland, Translational Research Institute (TRI), Brisbane, Australia; 4 Northern Ireland Centre for Stratified Medicine, University of Ulster, C-TRIC Building, Altnagelvin Hospital Campus, Glenshane Road, Londonderry, BT47 6SB UK; 5 Center for Cancer Research and Cell Biology, Queen’s University Belfast, Belfast, UK; 6 Thoracic Oncology Research Group, Institute of Molecular Medicine, Trinity College Dublin, Dublin, Ireland; 7 HOPE Directorate, St. James Hospital, Dublin 8, Ireland; 8 Department of Surgery, St Vincent’s Hospital Melbourne, University of Melbourne, Melbourne, VIC Australia

**Keywords:** Cell death, Cell growth, Cancer therapy, Lung cancer, Cancer, Cell biology

## Abstract

SASH1 (SAM and SH3 domain-containing protein 1) is a tumor suppressor protein that has roles in key cellular processes including apoptosis and cellular proliferation. As these cellular processes are frequently disrupted in human tumours and little is known about the role of SASH1 in the pathogenesis of the disease, we analysed the prognostic value of SASH1 in non-small cell lung cancers using publicly available datasets. Here, we show that low SASH1 mRNA expression is associated with poor survival in adenocarcinoma. Supporting this, modulation of SASH1 levels in a panel of lung cancer cell lines mediated changes in cellular proliferation and sensitivity to cisplatin. The treatment of lung cancer cells with chloropyramine, a compound that increases SASH1 protein concentrations, reduced cellular proliferation and increased sensitivity to cisplatin in a SASH1-dependent manner. In summary, compounds that increase SASH1 protein levels could represent a novel approach to treat NSCLC and warrant further study.

## Introduction

Lung cancer is the most commonly diagnosed cancer in the world (excluding non-melanoma skin cancers) and the fifth most common in Australia, where it is responsible for almost one in five cancer deaths^1^. The five-year patient survival rate of lung cancer is ~ 15%. Clearly, there is an unmet need for new therapeutic interventions, to improve the overall response rate and subsequent survival rate of lung cancer patients.

SAM and SH3 domain containing 1 (SASH1) was initially identified as a putative tumor suppressor gene, based on detection of significantly lower mRNA levels in lung, breast, thyroid and colorectal cancers compared to adjacent normal tissue^2,3^. SASH1 has previously been recognized as having a potential role in lung cancer susceptibility with the SASH1 gene located at a major lung cancer susceptibility locus on Chromosome 6q23-25^4,5^**.** Low mRNA expression of SASH1 correlates with poor prognosis in colon cancer^6^ and glioma^7^. In support of its role as a tumour suppressor, several recent studies have demonstrated that SASH1 opposes cancer cell proliferation, mesenchymal differentiation, migration and invasive cell behaviour in hepatocarcinoma, thyroid and cervical cancer cell lines^3,7–12^. Recent studies suggest that promoter hypermethylation correlates with SASH1 repression in tumours^13,14^. SASH1 localises to the nucleus, and its SAM and SH3 domains imply signalling, adaptor and/or molecular scaffold functions, although the precise molecular functions of SASH1 in normal tissues and cancer remain to be fully elucidated^15,16^. SASH1 can regulate signalling through focal adhesion kinase (FAK) and AKT/PI3K^3,10^ and promote apoptosis^3,8^. SASH1 has also been shown to be required for embryonic development by the regulation of alveolar epithelium cell maturation through nitric oxide signaling^17^.

Our previous work has demonstrated that SASH1 regulates apoptotic pathways and is cleaved by Caspase-3 during UVC-induced apoptosis^18^. Beyond its roles in tumorigenesis, SASH1 has also been demonstrated to be involved in the regulation of melanogenesis by a novel p53/POMC/α‐MSH/Gαs/SASH1 cascade. Highlighting this, mutations in SASH1 resulting in hyperpigmentation^19–21^.

Our previous data have shown that chloropyramine, a known competitive reversible H1-receptor antagonist (also known as an H1 inverse agonist), can increase SASH1 protein levels in breast cancer cells^22^. Chloropyramine exerts its pharmacological action by competing with histamine for the H1 subtype histamine receptor. Chloropyramine has also been shown to inhibit VEGFR-3 and FAK, showing anti-cancer activity in several tumor types, with a decrease in cell proliferation^23,24^.

The backbone of first-line treatment for most lung cancer patients, without identified mutations in genes such as EGFR, comprises combinational therapies using cytotoxic chemotherapeutic agents such as cisplatin^25^, which cause an overwhelming level of irreparable DNA damage. These are selectively cytotoxic to rapidly dividing cells directing them towards apoptotic cell death^26^. Despite a typical initial efficacy of these agents, NSCLC tumors eventually develop resistance. The resensitisation of these cancer cells to chemotherapeutics is key to improving patient survival. Here, we show that increasing SASH1 levels via exogenous overexpression or using a compound, could be a new strategy to treat NSCLC.

## Results

### SASH1 is an independent prognostic factor in NSCLC

**Figure 1:**
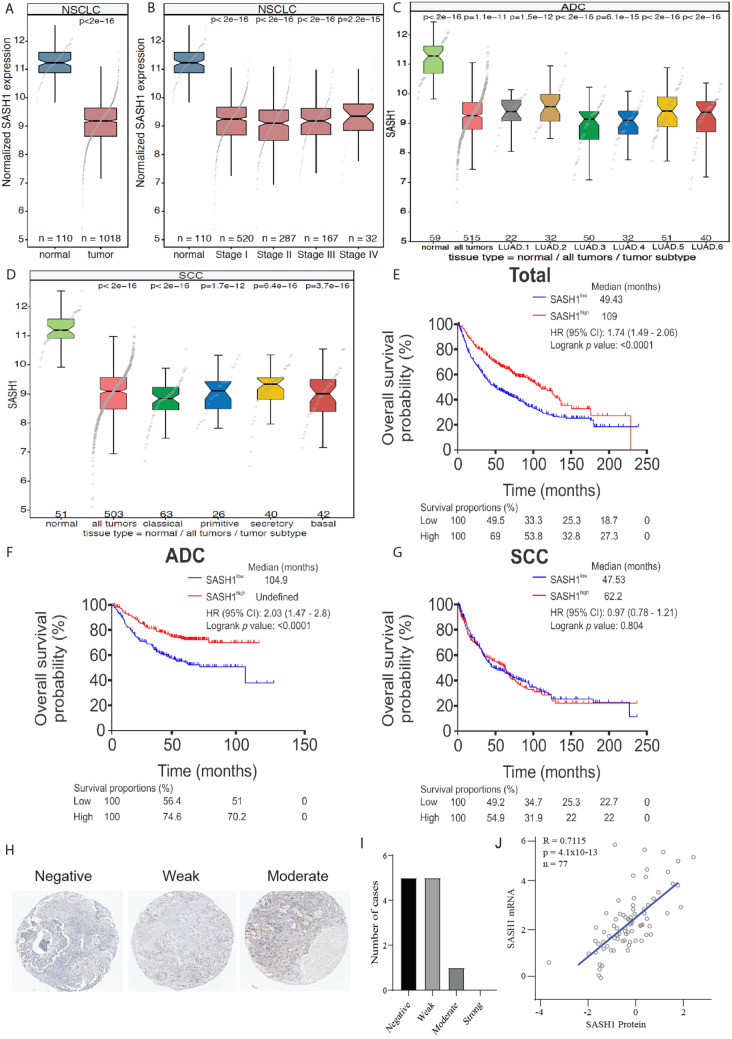
Reduced SASH1 mRNA expression is a prognostic indicator for poor lung cancer patient survival. (**A**–**D**) Box plots generated from The Cancer Genome Atlas (TCGA) RNAseq lung cancer datasets of overall SASH1 expression in lung tumor and normal tissue (**A**), stages of disease (**B**), adenocarcinoma subsets (**C**) and squamous cell carcinoma subsets (**D**). Shown are the median expression levels with 95% confidence intervals (notches), interquartile ranges (boxes) and all data points (grey dots). *P* values: Mann–Whitney *U* tests compared to expression in normal tissues. Sample sizes (n) are shown above the x-axes. (**E**–**G**) Univariate Kaplan–Meier analysis of overall survival using Medium expression. Overall survival was assessed in all NSCLC cases (**E**), adenocarcinoma (**F**) and squamous cell carcinoma (**G**). SASH1 protein expression was assessed using the protein atlas data, expression was shown to be low in lung carcinoma (**H**–**I**) using the same anti-SASH1 Antibody used in this study (HPA029947). SASH1 mRNA and protein levels were compared in 77 lung cancer cell lines (**J**). (**A**–**D**) were generated in the R computing environment (version 4.0, R Project for Statistical Computing, Vienna, Austria, https://www.r-project.org).

To determine whether SASH1 was a prognostic factor for NSCLC, SASH1 mRNA expression was assessed between normal and tumor tissue in a NSCLC cohort (Fig. 1A), and between stages of disease (Fig. 1B). SASH1 mRNA expression was evaluated separately in adenocarcinoma and squamous cell carcinoma cohorts. All subtypes examined showed decreased SASH1 expression in the tumour tissue compared to normal tissue (Fig. 1C,D). Kaplan–Meier survival analysis was performed for all 1145 patients stratified using medium SASH1 expression as the cut-off. Evaluation of all NSCLC cases in the cohort indicated there was a significant difference in survival based on SASH1 expression, with high SASH1 showing improved survival (Fig. 1E; HR (95% CI): 1.74 (1.49–2.06), *p* = 6.3 × 10^–11^ for low SASH1 versus high SASH1, log-rank *p* < 0.0001)). However, separate analysis of adenocarcinoma and squamous cell cohorts showed that this stratification was only statistically significant in the adenocarcinoma subset. SASH1 expression in squamous cell carcinomas did not stratify patients survival (Fig. 1F). In contrast, overall survival for patients with SASH1 high expressing adenocarcinomas was associated with improved outcomes (Fig. 1G; HR (95% CI): 2.03 (1.47–2.8), *p* = 2.0 × 10^–5^ for low SASH1 versus high SASH1, log-rank *p* < 0.0001). Further protein bioinformatic analysis from The Protein Atlas database demonstrated that low SASH1 protein expression is evident in the majority of cases, with 10/11 cases having weak or negative intensity (Fig. 1H,I). The correlation of SASH1 mRNA and protein was determined to have an R^2^ value of 0.7115 within a lung cancer cohort (Fig. 1J).

To assess if low SASH1 expression independently predicts poor patient survival, we performed multivariable survival analyses, which included stage, gender and smoking history as co-variates. Consistent with above univariate analyses, in multivariate Cox proportional hazard analyses, low SASH1 expression significantly predicts poor survival outcome for patients with NSCLC (*p* = 0.0092) and lung adenocarcinoma (p = 0.0095) but not for squamous cell carcinoma (*p* = 0.2815) (Table 1). Thus, this indicates that SASH1 is an independent prognostic factor for lung adenocarcinoma.

**Table 1:** Multivariate survival analysis of SASH1 in non-small cell lung cancer.

Cancer type	n	Variable	HR (95% CI)	*p* value	*p* value summary
NSCLC	441	Stage	2.13 (1.58–2.87)	< 0.0001	****
		Gender	1.39 (0.92–2.12)	0.1206	n/s
		Smoking history	0.77 (0.45–1.33)	0.3522	n/s
		SASH1	0.57 (0.37–0.87)	0.0092	**
		Model	0.42 (0.29–0.62)	< 0.0001	****
ADC	371	Stage	2.21 (1.56–3.13)	< 0.0001	****
		Gender	1.01 (0.63–1.64)	0.9546	n/s
		Smoking history	0.64 (0.36–1.12)	0.1161	n/s
		SASH1	0.53 (0.33–0.86)	0.0095	**
		Model	0.4 (0.26–0.63)	< 0.0001	****
SCC	59	Stage	1.92 (0.93–3.98)	0.0783	n/s
		Gender	3.5 (1.02–12.02)	0.0470	*
		Smoking history	9.05 (0.94–86.9)	0.0562	n/s
		SASH1	0.56 (0.2–1.6)	0.2815	n/s
		Model	0.41 (0.16–1)	0.0450	*

*ADC* lung adenocarcinoma, *CI* confidence interval, *HR* hazard ratio, *NSCLC* non-small cell lung cancer, *SCC* lung squamous cell carcinoma.

### Depletion of SASH1 increases the proliferation of NSCLC cells and confers cisplatin resistance

**Figure 2:**
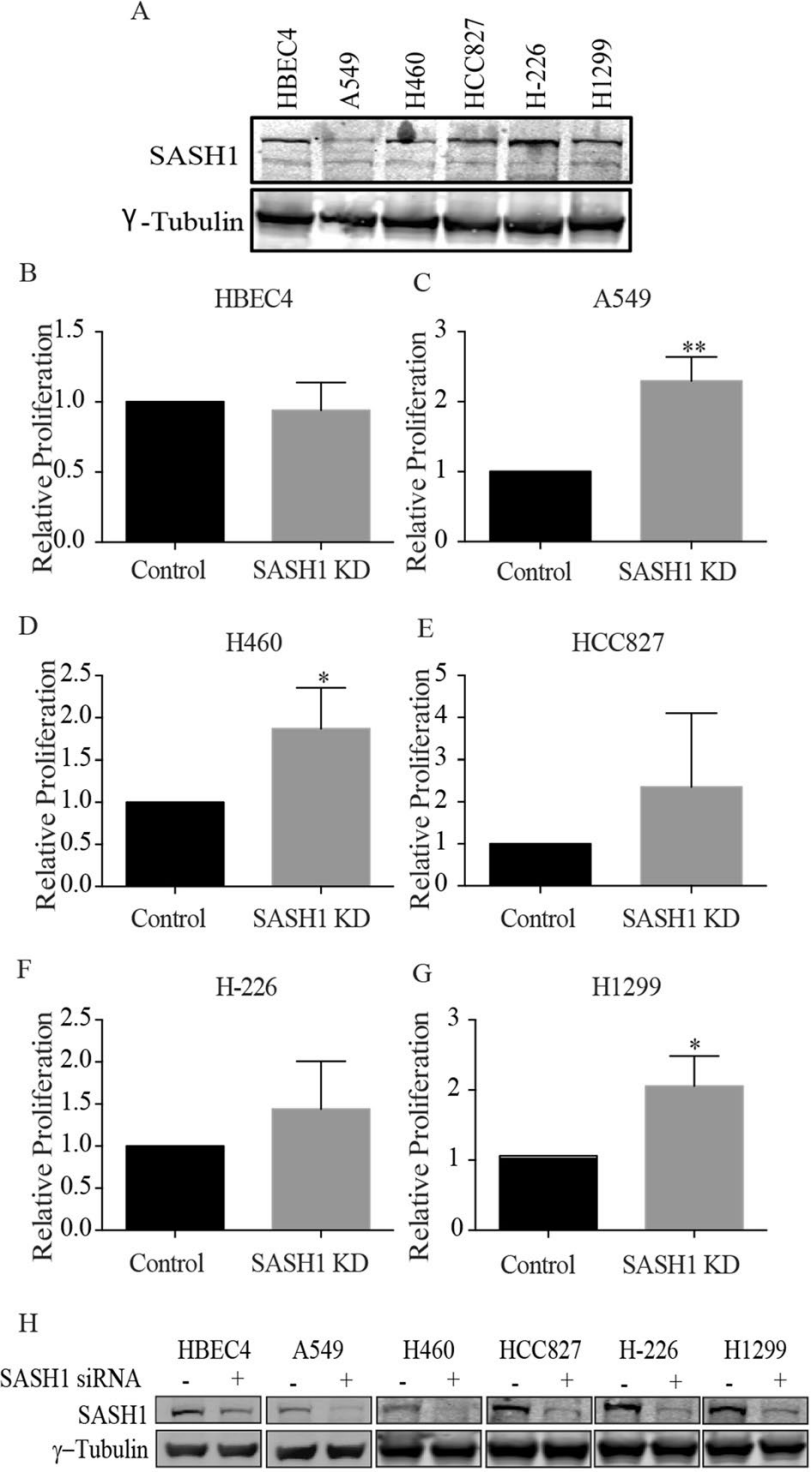
Lung cancer cell lines display increased proliferation following SASH1 depletion. (**A**) Immunoblot indicating SASH1 protein levels across panel of lung cancer cell lines. (**B**–**G**) SASH1 depletion with esiRNA in lung cells as indicated. Cell confluence was measured 72 h post SASH1 depletion. Data was normalised to control samples. (**H**) Immunoblot of SASH1 depleted lung cancer cells from (**B**–**G**) showing SASH1 depletion.

To determine whether SASH1 acts as a tumor suppressor in NSCLC, we next examined whether SASH1 depletion altered the proliferation of a panel of NSCLC cell lines. SASH1 depletion in the A549, H460 and H1299 NSCLC cell lines significantly increased cellular proliferation. An increase was also observed in HCC827 and H226 cancer cell lines but did not reach statistical significance (Fig. 2). No effect on cellular proliferation was observed in the non-tumorigenic HBEC cell lines (Fig. 2). This is consistent with other studies that have shown that depletion of SASH1 results in increased cellular proliferation in breast, lung, colon and ovarian cell lines^2,3,6,10,17,22^.

**Figure 3:**
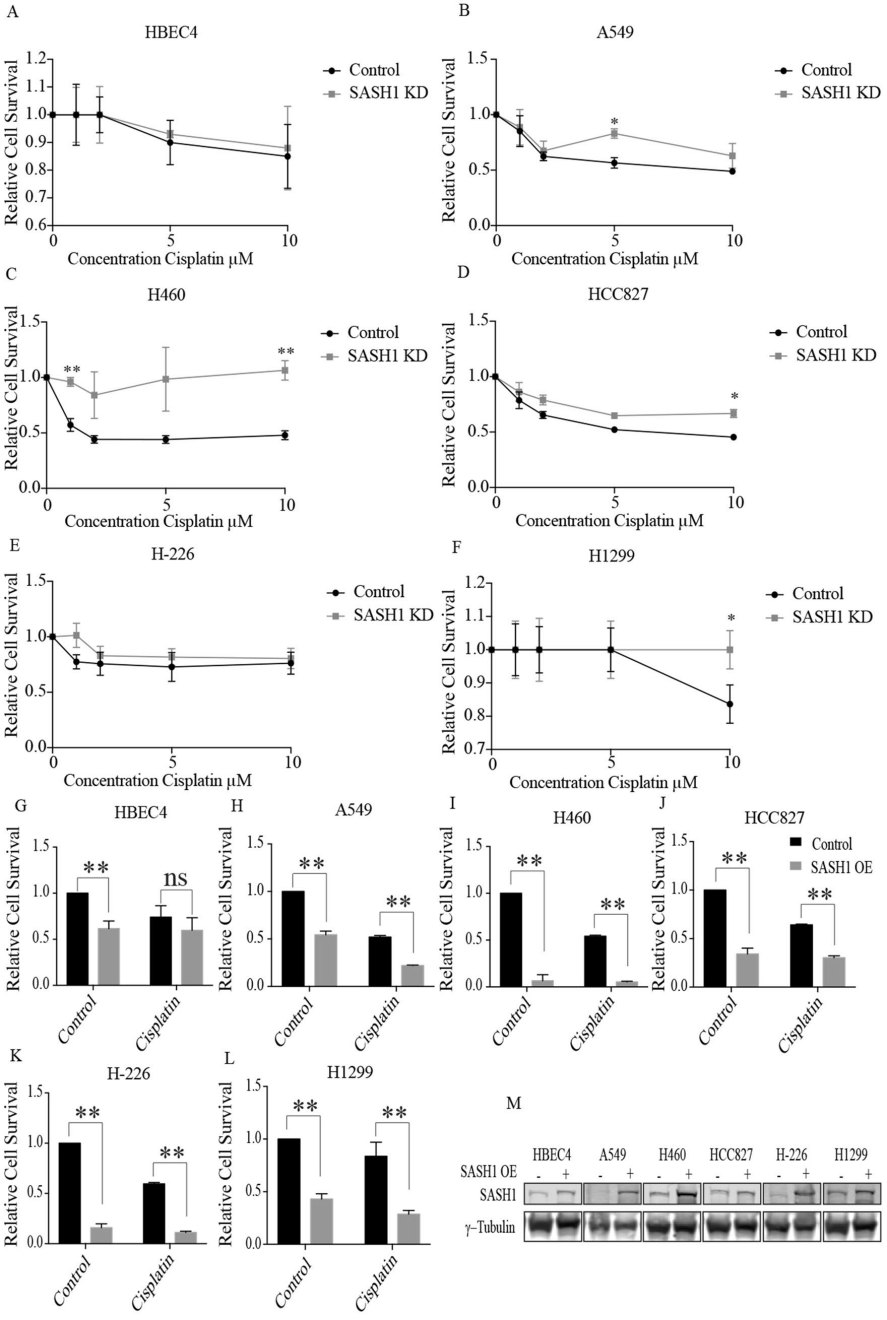
SASH1 protein levels can mediate cisplatin sensitivity. (**A**–**F**) SASH1 depletion with esiRNA in lung cancer cells confers resistance to cisplatin. Cell were seeded at equal density 48 h post depletion of SASH1 and treated with cisplatin at indicated doses (1–10 μM) 6 h post seeding. Cell survival was measured 48 h following cisplatin treatment. (**G**–**L**) SASH1 overexpression results in decreased cell proliferation with an additive effect from cisplatin treatment. Cells were transfected with SASH1-Flag or Flag alone (Control) and seeded 24 h post transfection cells where treated with cisplatin at IC30 concentrations 6 h post seeding. (**M**) Immunoblot of SASH1 overexpression in lung cancer cells from (**G**–**L**) indicating SASH1 expression.

As platinum-based chemotherapy is among the most utilised chemotherapeutic treatment strategies in lung cancer, we examined the impact of SASH1 depletion on the cellular sensitivity of NSCLC cell lines to cisplatin. As shown in Fig. 3A–F, the depletion of SASH1 led to increased cell survival suggesting that SASH1 may mediate cisplatin resistance. SASH1 depletion in HBEC cells did not affect sensitivity to cisplatin, indicating this effect may be specifc to tumour cells. Exogenous overexpression of SASH1 has been shown to increase cell death in several tumor cell lines^3,5–7^. To investigate this in NSCLC cell lines, Flag-SASH1 protein was transiently over-expressed (Fig. 3M). It was observed that overexpression of SASH1 in the NSCLC cell lines resulted in a significant decrease in cell survival, when compared to cells expressing the Flag vector alone (Fig. 3G–L). Ectopic expression of SASH1 also yielded a decrease in cell survival of NSCLC cells treated with cisplatin. This suggests that increasing SASH1 protein levels in NSCLC may be a strategy to reduced tumour cell proliferation.

### Chloropyramine treatment increases SASH1 protein expression and enhances NSCLC cell sensitivity to cisplatin

Previously, we hypothesised that pharmacologically increasing SASH1 levels may be a novel approach to cancer therapy. To identify compounds that increased SASH1 protein levels we performed connectivity mapping using the CMap database (Broad Institute^27^). This identified a direct correlation between chloropyramine treatment and SASH1 mRNA expression (p = 0.000005, z-score 2.431). Our earlier work in breast cancer indicated that chloropyramine can induce cell death via a SASH1-dependent mechanism^22^.

**Figure 4:**
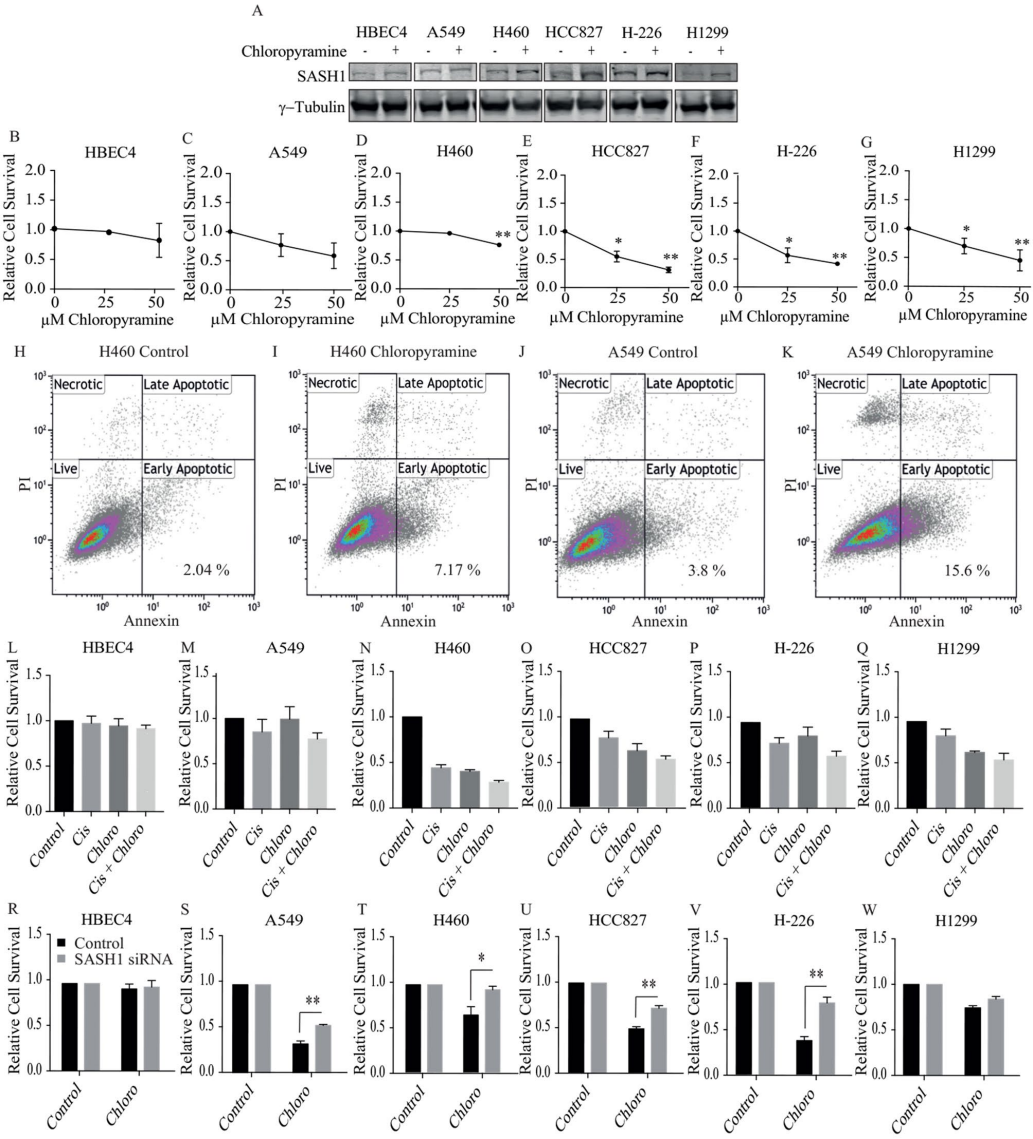
Chloropyramine increases SASH1 levels in lung cancer cells causing increase in cell death in combination with cisplatin. (**A**) Immunoblot of lung cells following 16 h treatment with 50 μM chloropyramine indicating an increase in SASH1 protein level. (**B**–**G**) Treatment of lung cancer cells with chloropyramine decreases cell survival in a dose dependent manner. Cell survival measured 48 h post chloropyramine treatment at either 0, 25 μM or 50 μM. (**H**–**M**) Lung cancer cells treated with cisplatin (IC30, (1–10 μM)) and chloropyramine (IC30 (10–50 μM)) have an additive reducing in cell survival. (**N**–**S**) Depletion of SASH1 protects cells from the anti-tumour effects of chloropyramine. Relative cell survival of SASH1 depleted cells in response to chloropyramine treatment. (**T**–**W**) Chloropyramine induces cell death in lung cancer cell lines. Cells were stained with propidium iodide and an Annexin V-FITC antibody conjugate 48 h post-chloropyramine treatment, and analysed by flow cytometry.

Here, we assessed the use of chloropyramine as a chemical agent to increase SASH1 protein levels and induce tumour cell killing of lung cancer cells. Treatment of NSCLC cells with chloropyramine increased SASH1 protein expression (Fig. 4A) and a significant decrease in cell survival at 25 μM for the HCC827, H-226 and H1299 cell lines and at 50 μM for all other NSCLC cell lines, with the exception of A549 cells (Fig. 4B–G). Interestingly, HBEC4 cells showed only a minor decrease in cell number at the higher dose threshold. The cells treated with chloropyramine were confirmed to be undergoing apoptosis via Annexin V and PI staining (Fig. 4H–K). The effect of cisplatin combined with chloropyramine was next examined in the HBEC and NSCLC cell lines. As shown in Fig. 4L–Q, the combination treatment with cisplatin and chloropyramine enhanced the induction of cell death in all NSCLC cell lines, compared with the cell death induced by either drug alone with the exception of the A549 and HBEC cells. Taken together, these data suggest that chloropyramine is effective at inducing cell death in lung cancer cells.

To determine whether the chloropyramine-induced cell death was SASH1-dependent, NSCLC cells were depleted of SASH1 using a targeted siRNA prior to treatment with chloropyramine (Fig. 4R–W). This demonstrated that SASH1 depletion partially rescued the cell death response in these cell lines, suggesting chloropyramine-induced cell death is at least in part dependent upon the function of SASH1.

## Discussion

SASH1 has been proposed as a tumor suppressor, based on the correlation of the presence of SASH1 mRNA expression with beneficial prognosis in several human cancers^2–8,28–31^ and the observation that loss or depletion of SASH1 enhances tumor cell line survival and invasiveness in vitro^3,5–8^. Our investigation of SASH1 mRNA expression in tumors has supported this data. However, in contrast to what we have observed here, our recently published work in breast cancer showed a strong association between high nuclear SASH1 protein expression and favorable outcome in ER + cases, suggesting that the prognostic impact may be context dependent^22^.

Here, we observed that overexpression of SASH1 at the protein level through exogenous gene transfection induced cell death. In contrast, downregulation of SASH1 using siRNA increased cellular proliferation, consistent with other SASH1 studies in malignant disease. We have previously described that the impact of SASH1 expression on proliferation of breast cancer cell lines occurs irrespective of the histological subtype^22^. Furthermore, similar observations have been made for one previous study in lung cancer and in a range of solid tumors including cervical, hepatocellular and thyroid carcinomas and osteosarcoma^3,9–12,15^. Although only a moderate difference in SASH1 protein expression was observed between the non-cancerous cell line HBEC4 and the lung cancer cell lines in our study, 5/11 lung cancer cases from the Human Protein Atlas database have non-detectable SASH1 protein levels. This supports the notion that SASH1 protein expression is regularly lost in lung tumours (Fig. 1H,I). As a novel observation we identified that low SASH1 expression is associated with resistance to cisplatin, whilst high SASH1 expression induces sensitivity to the chemotherapeutic agent.

One question that arises is whether or not SASH1 protein levels can be induced pharmacologically. Using connectivity mapping we previously identified chloropyramine as a potential inducer of SASH1 expression^22^. In our previous study, we identified that this agent induces cell death in a SASH1 dependent manner, increasing protein levels within the cells. In the present study we have made similar observations, suggesting that increasing SASH1 levels may also be a viable therapeutic strategy in NSCLC. The repurposing old agents as cancer therapeutics is gaining pace, as our understanding of the mode of action of these agents increases. The potential introduction of chloropyramine into cancer treatment regimens still requires extensive investigation. Chloropyramine is currently approved in some European countries as an anti-histamine agent and has the advantage of minimal side effects. The further investigation of the mechanism by which chloropyramine increases SASH1 level will allow the development of new-targeted agents to increase the efficacy of targeting SASH1 as a cancer therapy.

Although changes to SASH1 expression have been linked with several other diseases, these associations need further investigation to cement the role of SASH1 as a tumor suppressor relevant to cancer prognoses, particularly within the context of apoptosis, cell cycle and proliferation. In support of this, our study has demonstrated that SASH1 does function within the cancer phenotype, with a prominent role in apoptosis. Agents that upregulate SASH1 are potential novel approaches to the management of lung cancer and warrant further preclinical and clinical studies of chloropyramine.

## Methods

### Cell culture and transfection

Cell lines were cultured at 37°C with 5% CO^2^ in media as outlined in Table 2. Chloropyramine (Sigma-Aldrich) was added to adherent cultured cells 24 h after seeding at the indicated concentrations (0–100 μM). Cancer cell lines include Adenocarcinoma (A549, H1299 and HCC827), Large cell carcinoma (H460) and Squamous cell carcinoma (H-226). All cell lines were originally sourced from American Type Culture Collection (ATCC).

**Table 2:** Cell line culture conditions.

Cell line	Type	Media
HBEC4	Transformed lung cell line	KSFM (0.2 ng/mL EGF, BPE 30 ug/ml)
H460	Large cell carcinoma	RPMI with 10% FCS
HCC827	Adenocarcinoma	RPMI with 10% FCS
H1299	Adenocarcinoma	RPMI with 10% FCS
H-226	Squamous cell carcinoma	RPMI with 10% FCS
A549	Adenocarcinoma	Ham’s F12 with 10% FBS

*RPMI* Roswell Park Memorial Institute Medium, *KSFM* Keratinocyte-SFM, *FCS* Fetal Calf Serum, *EGF* Epidermal growth factor, *BPE* Bovine Pituitary extract.

siRNA experiments where performed as previously described^19^, briefly esiRNAs (Sigma) targeting SASH1 or non-specific control oligos (20 nM) were transfected using RNAiMax (Invitrogen) as per the manufacturer's instructions. Double-transfections were performed 24 h apart and samples were analysed 72 h post the initial transfection, where optimal SASH1 depletion was observed. For overexpression studies, the full-length SASH1 cDNA was cloned into the mammalian expression vector pCMV6 (Origene). 3 μg of DNA (SASH1-Flag or Flag) and 8 μL of Fugene HD were used to transfect cells in a T25 flask, as per the manufacturer's instructions. Cells were assayed 48 h post-transfection for optimal overexpression and death assessment.

### Immunoblotting

Immunoblotting was carried out as described previously^32^. Uncropped blots are provided in the supplementary data file.

### Cell death assay

Following incubation of cells with the indicated treatments, propidium iodide (10 μg/ml) and Hoechst (1 μg/ml) were added 30 min before imaging. Cells were imaged on an IN Cell Analyzer 2200 (GE Healthcare; 10 × objective). Live/dead cell analysis was performed using IN Cell analysis software.

Apoptosis was assessed using Annexin PI staining as per Promega Annexin V-FITC apoptosis detection kit instructions.

### Cell proliferation assay

Control or SASH1 depleted lung cancer cells were seeded at 2500 cells per well in 96 well plates (Nunc). Cells adhered and then imaged every 2 h for 96 h in an IncuCyte ZOOM live cell imager (Essen Bioscience) to calculate confluence.

### Drug screen with connectivity mapping

An established gene expression connectivity mapping approach^33,34^ was employed to screen candidate drugs for their potential to induce SASH1 expression. Chloropyramine was identified as an agent associated with upregulation of SASH1 expression as previously described in breast cancer^22^.

### Bioinformatics analyses

Figure 1A–D were generated using The Cancer Genome Atlas (TCGA) lung cancer RNAseq and clinical datasets^35,36^. For this, levels 3, log2-transformed mRNA expression levels and associated clinical files were downloaded from the NIH-NCI Genomic Data Commons Data Portal (https://portal.gdc.cancer.gov) for both adjacent normal (henceforth "normal") and lung tumor samples. Samples from the lung adenocarcinoma (LUAD) and lung squamous cell carcinoma (LUSC) were considered separately, as well as combined. The latter comprised the non-small cell lung cancer (NSCLC) dataset. Statistical analyses and generation of plots were performed in the *R* statistical environment (version 3.6, *R* Core Team, Vienna, Austria). The *p* values were calculated using the (unpaired) Mann–Whitney *U* test, all relative to normal tissue. They have not been adjusted to account for multiple hypothesis testing. Kaplan–Meier survival curves (Fig. 1E–G) were generated from Kaplan–Meier plotter using medium expression cutoff with multivariate analysis performed. Survival analyses were performed using the SASH1 microarray probe 226022_at. Statistical analyses of survival curves included log-rank tests, with ***p*** values reported, as well as univariate and multivariate Cox proportional hazard analyses, with hazard ratios (HR) and 95% confidence intervals (CI) reported. In addition, for the SASH1^**low**^ and SASH1^**high**^ groups, survival proportions at 50-month intervals and median survival in months were reported. SASH1 mRNA and protein levels were compared in 77 lung cancer cell lines. Normalized log2-transformed RSEM mRNA levels, in transcripts per million (TPM), were obtained from reference^31^. Protein levels were determined by mass spectrometry and represent normalized quantities obtained from reference^37^. Linear regression and Spearman correlation analyses were used to determine the mRNA-protein correlation in the cell lines. SASH1 protein expression was investigated in a tumor cohort utilising data from The Human Protein Atlas. Data from the SASH1 Antibody HPA029947 was used for the data presented^38,39^.

### Statistical analysis

Cell proliferation analysis was carried by Graph Pad Prism, with T Test used to assess differences between subsets. Biological replicates performed as indicated with P values where assigned *P < 0.05, **P < 0.005 and ***P < 0.0005.

## Supplementary information

Supplementary information
