# Supplementary material for: SASH1 is a prognostic indicator and potential therapeutic target in non-small cell lung cancer
Source: Sci Rep. 2020 Oct 29;10:18605. doi: 10.1038/s41598-020-75625-1 (PMC7596716; doi:10.1038/s41598-020-75625-1)

SASH1 is a prognostic indicator and potential therapeutic target in non-small cell lung cancer – Supplemental data

**Joshua T. Burgess ^1*^, Emma Bolderson ^1,2*^, Mark N. Adams ^1^, Pascal H.G. Duijf ^1,3^, Shu-Dong Zhang ^4,5^,**

**Steven G. Gray ^6,7^, Gavin Wright ^8^, Derek J. Richard ^1^, Kenneth J. O’Byrne ^1,2^*.**

^1^ Cancer & Ageing Research Program, Institute of Health and Biomedical Innovation at the Translational Research Institute (TRI), Queensland University of Technology, Brisbane, Australia.

^2^ Princess Alexandra Hospital, Ipswich Road, Woolloongabba, Brisbane, Queensland 4102, Australia.

^3^ University of Queensland Diamantina Institute, The University of Queensland, Translational Research Institute (TRI), Brisbane, Australia.

^4^ Northern Ireland Centre for Stratified Medicine, University of Ulster, C-TRIC Building, Altnagelvin Hospital campus, Glenshane Road, Londonderry, BT47 6SB, UK.

^5^ Center for Cancer Research and Cell Biology, Queen’s University Belfast, United Kingdom.

^6^ Thoracic Oncology Research Group, Institute of Molecular Medicine, Trinity College Dublin, Ireland.

^7^ HOPE Directorate, St. James Hospital, Dublin 8, Ireland.

^8^  University of Melbourne Department of Surgery, St Vincent's Hospital Melbourne, Victoria, Australia.

***** Correspondence:

Dr. Joshua Burgess, [j6.burgess@qut.edu.au](mailto:j6.burgess@qut.edu.au), (+61 (0) 405979589)

Dr. Emma Bolderson, [emma.bolderson@qut.edu.au](mailto:emma.bolderson@qut.edu.au) (+61 (0) 422770649)

Prof. Kenneth O’Byrne, [ken.obyrne@qut.edu.au](mailto:ken.obyrne@qut.edu.au) (+61 (0) 449091958)

Address: 37 Kent Street Woolloongabba, 4102 Brisbane Australia, Fax +61 (7) 34437236

Figure 2 a:


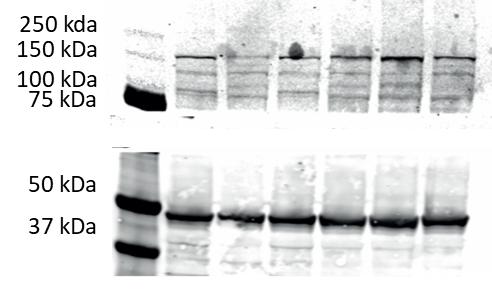


Figure 2 H


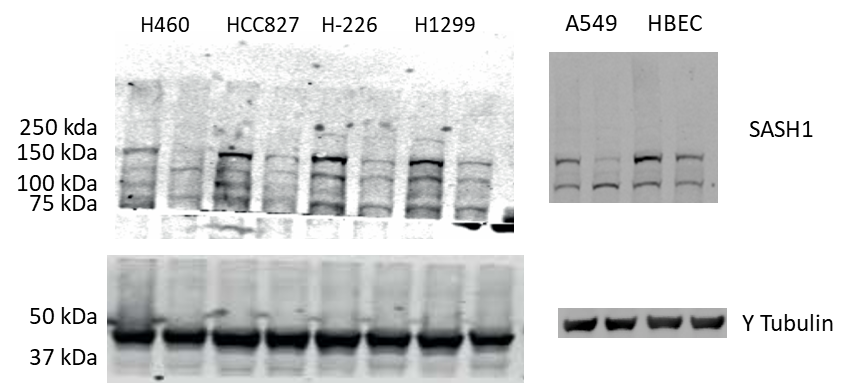


Figure 3 M


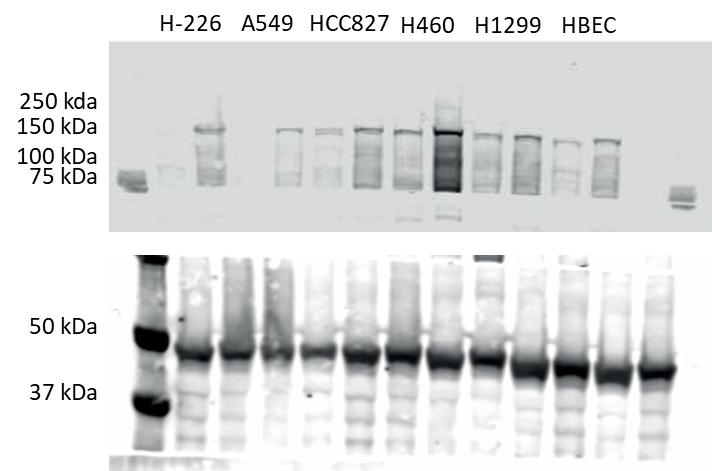


Figure 4 A:


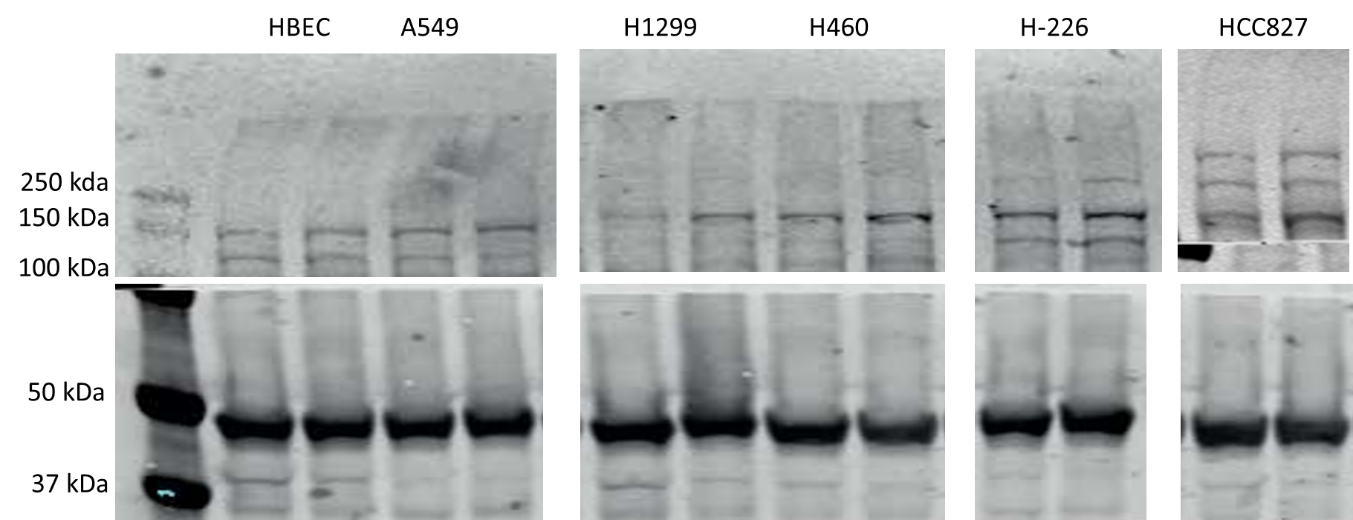

Supplement: Supplementary file 1 — Supplementary information [file 41598_2020_75625_MOESM1_ESM.docx]
